# Bilateral Retrobulbar Optic Neuritis Caused by Varicella Zoster Virus in a Patient with AIDS

**DOI:** 10.9734/BJMMR/2015/14259

**Published:** 2014-11-11

**Authors:** Jose F. Duda, Jose G. Castro

**Affiliations:** 1 Employee Health Unit, International Committee of the Red Cross, Geneva, Switzerland.; 2 Division of Infectious Diseases, University of Miami Miller School of Medicine, Florida, United States of America.

**Keywords:** Optic neuritis, varicella-zoster virus, HIV, AIDS, retinal diseases

## Abstract

**Aims:**

To report on a case of bilateral retrobulbar optic neuritis in a patient with acquired immune deficiency syndrome (AIDS) caused by varicella-zoster virus (VZV); and to review the literature focusing on: cases reported, epidemiology, pathophysiology, diagnosis and treatment.

**Presentation of Case:**

A 38-year-old woman with AIDS presented with a 10-day history of progressive bilateral visual loss and ocular pain. She had bilateral dilated pupils with no light perception; the fundoscopic examination was normal. Facial herpes zoster lesions appeared on the second day of hospitalization Magnetic resonance imaging (MRI) findings were compatible with a bilateral optic neuritis; the cerebrospinal fluid (CSF) showed pleocytosis, increased proteins and a positive VZV-DNA PCR. She was treated with intravenous acyclovir and corticosteroids and was able, when discharged 2 weeks after admission, to carry out activities of daily living.

**Discussion:**

VZV retrobulbar optic neuritis has previously been reported in 12 patients with AIDS, more than half of the cases had concomitant herpes zoster and an associated retinopathy. A positive VZV-DNA in the CSF is indicative of VZV infection, initial use of intravenous acyclovir is recommended, and the concomitant use of corticosteroids would be a prudent choice; the duration of antiviral therapy remains undefined.

**Conclusion:**

VZV retrobulbar optic neuritis in AIDS patients can occur with or without herpes zoster. It is a sight-threatening infectious and inflammatory process requiring the advice of specialists in infectious diseases, ophthalmology, neurology and viral microbiology.

## 1. INTRODUCTION

VZV is a highly communicable herpesvirus and in the prevaccine era serologic evidence of previous infection was present in 90% of persons by the age of 15 [1]. The primary infection causes a systemic febrile illness followed by a latent infection within the sensory ganglia, the reactivation causes herpes zoster (shingles). In the prevaccine era 0.1 % of VZV infections develop in the immunosuppressed hosts but this group accounted for as many as 25 percent of varicella-related deaths [2].

Herpes zoster ophthalmicus affects the ophthalmic division of the trigeminal nerve, is associated with severe complications and the risk for human immunodeficiency virus (HIV) positive patients is 6.6 higher [1]. The ocular manifestations of VZV infection include dermatitis, keratitis, uveitis, retinitis and optic neuritis. Infectious retrobulbar optic neuritis caused by VZV is rare but well documented, it can present with or without concurrent VZV cutaneous lesions and can lead to vision loss [1,2], it has been reported in HIV negative [3-5] and HIV positive patients [6-14]. We describe a case of a patient with AIDS and bilateral retrobulbar optic neuritis, the visual symptoms preceded the appearance of trigeminal herpes zoster and the fundoscopic exam was normal. We compare the findings with the literature.

## 2. PRESENTATION OF CASE

A 38-year-old woman was transferred to our hospital because of decreased visual acuity, with inability to perceive light in both eyes. She had a history of headache, ocular pain and blurred vision in both eyes for 10 days, the visual acuity decreased progressively, two days before admission she required assistance to walk and on the day of admission she was unable to perceive light. She had a history of untreated HIV infection for 7 years; her absolute CD4 cell count was 15 cells/uL and serum HIV RNA level was 169,000 copies/uL.

The physical examination was remarkable for underweight with a body mass index of 18 kg/m2, temporal wasting and oral thrush. She had dilated pupils with no light perception bilaterally, eye movements were normal but painful. The corneas and fundoscopic examination were normal. The rest of the physical exam, including a detailed neurologic examination, was normal. CT scan of the brain with contrast showed mild brain atrophy. Initial lumbar puncture disclosed an opening pressure of 34 cm H_2_O. The CSF was clear with 16 white blood cells/uL, 14 neutrophils and 2 lymphocytes, normal glucose level and protein level of 74 mg/dL. Results of VDRL, cryptococcus antigen, acid-fast and Gram stains were negative.

One day after admission, a vesicular rash was noted on the left side of her face involving the lips, bridge of the nose, and eyelids. Acyclovir 10 mg/kg intravenously every eight hours and prednisone 1 mg/kg were started. MRI with gadolinium of the brain and optic nerves showed diffuse bilateral enhancement of the optic nerves.

The results of CSF PCR reaction for *Mycobacterium tuberculosis*, Epstein-Barr virus, herpes simplex virus 1 and 2, cytomegalovirus and John Cunningham polyomavirus were negative, VZV-DNA PCR was positive. Cultures for viruses, fungi, bacteria and mycobacteria remained negative.

One week after admission the patient was no longer requiring opiate analgesics for the headache, there was light perception and a second lumbar puncture showed: normalization of the opening pressure, no cells, normal glucose level, total protein level of 49 mg/dL and the DNA PCR for VZV was still positive. The patient's vesicular rash resolved in 10 days.

The patient was discharged 2 weeks after admission on oral acyclovir. She was able to carry out activities of daily living without assistance. She did not returned to the outpatient clinic for follow up.

## 3. DISCUSSION

We have presented the case of a woman with AIDS in whom bilateral retrobulbar optic neuritis developed as a result of VZV infection. We discuss below the epidemiology, pathophysiology, diagnosis and treatment.

In 1943 Ruska reported the VZV in electron microscopic studies of chickenpox vesicle fluids, ten years later Weller induced similar cytopathic changes from vesicular fluids of chickenpox and shingles in culture [2]. VZV is an enveloped double-stranded DNA herpesvirus and a member of *Alphaherpesviridae* [1].

Herpes zoster usually presents as a painful cutaneous vesicular eruption in a dermatomal distribution and the cranial nerve dermatomes are involved in 20%-25% of cases [15]. However, VZV complications can occur without a preceding episode of shingles, a condition known as *zoster sine herpete* [1]. The ophthalmic division of the trigeminal nerve is involved in 10%-17.5% of cases and 50%-89% of those cases will present ocular complications [1]. Optic neuritis and necrotizing retinopathy are known complications described in immunocompetent and immunocompromised patients [15].

The risk of herpes zoster is higher in HIV-seropositive patients, a cohort study on homosexual men, 287 HIV-seropositive and 499 HIV-seronegative showed an incidence of 29.4 cases/1000 person-years and 2 cases/1000 person-years, respectively [16]. VZV retrobulbar optic neuritis is a rare presentation and it has been reported in immunocompetent and immunocompromised HIV-seronegative patients [3-5]. In HIV-seropositive patients the 12 cases reported in the literature had AIDS [6-14].

The histopathology of VZV optic neuritis shows demyelination with mononuclear cell infiltration and intranuclear inclusions [3,17,18]. Necrosis of the optic nerve has also been described with swollen endothelial cells and cellular thrombi of the branch arteries [18]. The compression of the swollen nerve in the optic canal probably amplifies the ischemic process.

Optic neuritis usually presents with headache and/or eye pain followed by a variable degree of visual loss (scotoma) affecting mainly central vision. An afferent pupillary defect is present if the lesion is unilateral or asymmetric and the fundoscopic examination shows absence of optic disc involvement in retrobulbar optic neuritis. The differential diagnosis of optic neuropathy in AIDS patients includes: central nervous system lymphoma, cryptococcus, cytomegalovirus, hepatitis B virus, histoplasmosis, HIV itself, syphilis and VZV [9,14].

The twelve HIV-seropositive cases with VZV retrobulbar optic neuritis reported in the literature had AIDS [6-14], in 5 cases there was no previous history suggestive of shingles or chickenpox, in 7 cases herpes zoster preceded or appeared shortly after the visual symptoms like in our case. Our case had bilateral eye involvement, in 5 of the 12 cases previously reported the disease progressed towards bilateral eye involvement. Retinopathy was documented at diagnosis in 4 cases and developed during the course in 6 of the 12 cases. Retinal detachment occurred in half of the 12 cases, this complication has been reported in 75% of patients with VZV retinitis [8].

VZV retrobulbar optic neuritis may precede a necrotizing retinopathy [8,17]; it may also occur afterwards or simultaneously [18]. The retinal necrosis is caused by an occlusive vasculopathy [17], and can develop 10-68 days after the diagnosis of optic neuropathy [9]. Necrotizing retinopathy caused by VZV can present as acute retinal necrosis (ARN) or progressive outer retinal necrosis (PORN) in immunocompetent and immunocompromised patients but PORN occurs almost exclusively in HIV-seropositive patients with CD4 cell count < 100 cells/uL [15]. Optic nerve involvement in patients with ARN has been reported in 47% to 57% of cases [18].

**Fig. 1:**
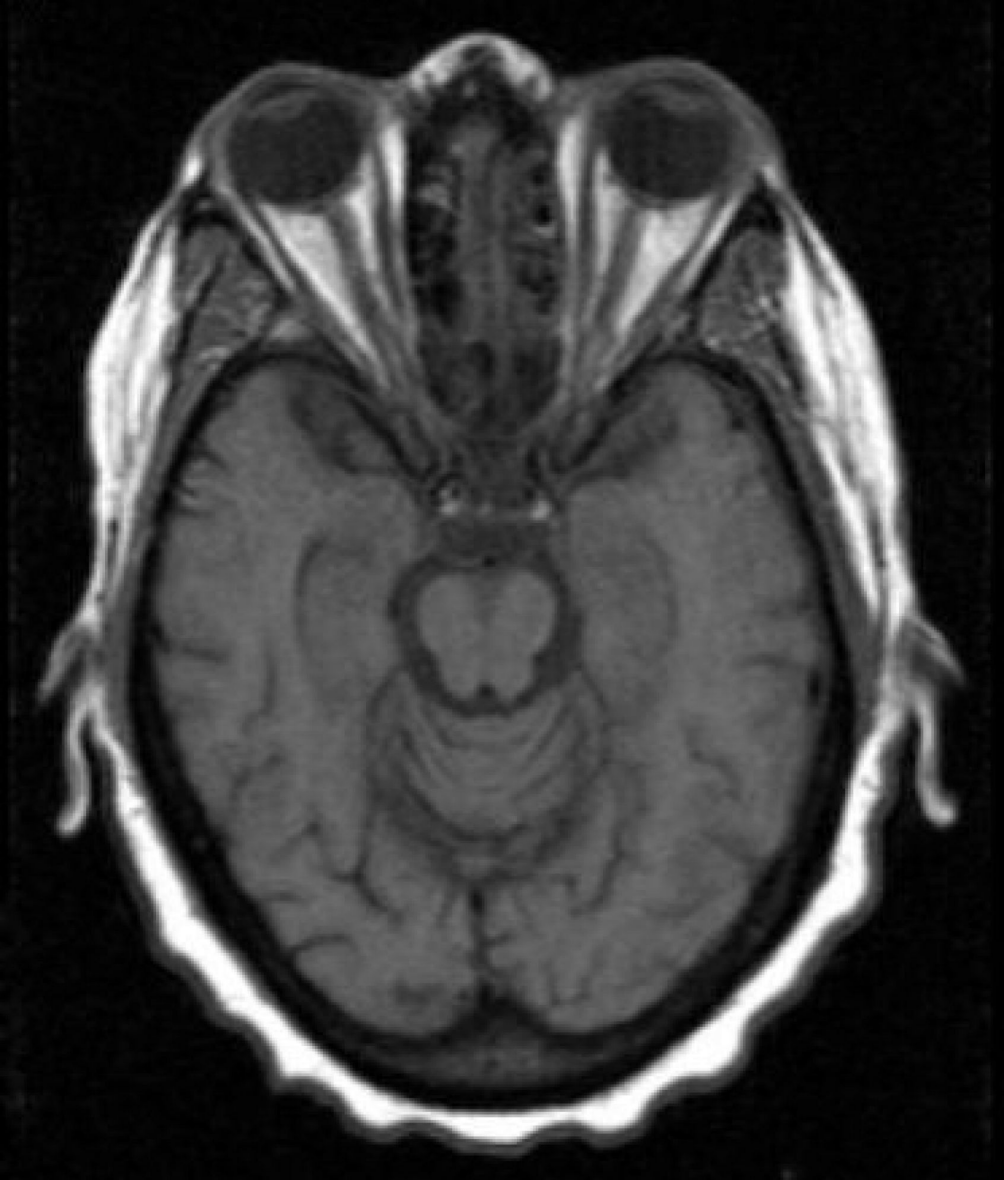
The axial T1-weighted MRI after intravenous injection of gadolinium shows marked signal enhancement of both optic nerves, indicating disruption of the blood-nerve barrier and optic neuritis

Herpes zoster can usually be diagnosed clinically, when the diagnosis is uncertain, swabs from a fresh lesion or tissue biopsy, can be submitted for culture, direct fluorescent antibody or PCR [15]. In the absence of lesions the diagnosis of VZV retrobulbar optic neuritis should be pursued in a patient with visual loss, normal fundoscopic examination and AIDS [8]. MRI of the brain and orbits with gadolinium contrast could demonstrate optic nerve inflammation (Fig. 1), nevertheless previous reports of VZV retrobulbar optic neuritis have shown normal or equivocal MRI findings [7,8,13,14].

A positive CSF analysis for VZV-DNA PCR or anti-VZV IgG is consistent with VZV infection of the central nervous system [17], in the context of visual loss in an AIDS patient it is indicative of VZV optic neuritis. The CSF VZV-DNA was positive in the five cases that reported the results of PCR analysis [7,8,9,12]. However, in a prospective study of 31 consecutive HIV infected patients undergoing necropsy, VZV DNA was detected in the CSF of 4 patients who did not have antemorten or necropsy evidence of ocular brain or spinal cord disease [19], which suggest that evidence of VZV replication is not always associated with clinical disease.

Intravenous acyclovir remains the treatment of choice in immunosuppressed patients with VZV neurological complications [1,2,15,17]. The recommended dose is 10 mg/kg three times daily for 10-14 days. Optic neuritis associated with VZV vaccine was recently reported in two cases [20], the authors reported neither the immune status of the patients nor the association with wild-type or vaccine VZV. Both cases made a complete recovery after treatment with high-dose intravenous corticosteroids. Based on the inflammatory histopathologic findings [3] it seems reasonable to use corticosteroids, nevertheless there are no reliable data to support their use.

Intravenous foscarnet is recommended to treat patients with proven or suspected acyclovir-resistant VZV [15]. Intravenous acyclovir followed by 6-8 weeks of oral antiviral therapy and optic nerve sheath decompression have been used in some patients with VZV “acute retinal necrosis associated optic neuropathy” [18], owing to the limited number of cases it is difficult to determine the efficacy of these approaches.

Because initiation of antiretroviral therapy (ART) has been associated with VZV mediated vasculitis in the context of central nervous system – immune reconstitution inflammatory syndrome [21] and because the risk of herpes zoster increases two to fourfold from baseline between 4-16 weeks after initiation of ART [15], it seems prudent to wait at least 16 weeks after the acute presentation of VZV optic neuritis to initiate ART.

## 4. CONCLUSION

Retrobulbar optic neuritis caused by VZV is a rare presentation in immunocompromised and immunocompetent patients, in HIV-seropositive patients have been reported exclusively in patients with AIDS; we believe that our case is the thirteenth reported. Retinopathy was documented on admission or during the course of the disease in the majority of patients and retinal detachment is a common complication.

VZV retrobulbar optic neuritis can occur with or without herpes zoster lesions. Diagnosis and management are challenging and requires the involvement, at an early stage, of specialists in infectious diseases, ophthalmology, neurology and viral microbiology.

The optimal treatment has not been defined. Because the optic neuritis is sight-threatening and the histopathology demonstrates inflammatory infiltrates, it seems reasonable to use intravenous acyclovir and corticosteroids initially.

It seems prudent to delay initiation of ART because it has been associated with VZV neurological complications and an increased risk of herpes zoster.

## CONSENT

Written informed consent was obtained from the patient for publication of this case report, personal identifiers were not included.
